# Surgical site infections after caesarean section across sub-Sahara Africa: a scoping review of prevalence and associated factors

**DOI:** 10.3389/fgwh.2025.1605049

**Published:** 2025-07-01

**Authors:** Rebekah Wood, Anna Borodova, Sophie Wolter, Micheline N’Guessan, Amadou Aziz Diallo, Mamadou Kamis Diallo, Katharina Heldt, Carlos Rocha, Ibrahima Nabé, Bamourou Diané, Mahamoud Sama Cherif, Sophie Alice Müller

**Affiliations:** ^1^ Centre for International Health Protection, Robert Koch Institute, Berlin, Germany; ^2^ University Hospital Bouaké, Bouaké, Côte d'Ivoire; ^3^ Faranah Regional Hospital, Faranah, Guinea; ^4^ Methods Development, Research Infrastructure and Information Technology, Robert Koch Institute, Berlin, Germany

**Keywords:** surgical site infection, wound infection, caesarean, sub-Saharan Africa, scoping review

## Abstract

Surgical site infections are among the most common healthcare associated infections worldwide and pose a significant risk in cesarean section procedures, which are the most frequently performed surgical operations globally. Our scoping review aimed to synthesize available literature from studies conducted in sub-Saharan Africa on the prevalence of post-caesarean surgical site infections, as well as on reported associated factors. In this scoping review we included studies retrieved in OVID from January 2014 to January 15, 2024 without restrictions on the language of publication or publication status focusing on studies from sub-Saharan African countries. The review was registered on the Open Science Framework platform. The reported rates of surgical site infection across the 73 included studies ranged from 2.0%–56.0%. A forest plot showed that the studies were highly heterogeneous, whereby only 11.0% showed a surgical site infection rate above 20.0%. Most surgical site infections surfaced within two-weeks after a caesarean section. The strongest and most frequently cited risk factors were: duration of labour ≥8 h, surgical duration, multiple vaginal examinations, stored water usage, and premature rupture of membrane. The following protective factors were also determined: Pfannenstiel/transversal incisions, caesarean section at term, having health insurance, normal body mass index, <1 h of surgical intervention, <24 h of premature rupture of membrane, low intraoperative blood loss and absorbable sutures. Staphylococcus *aureus* was the most commonly isolated pathogen among studies with bacteriological reporting. Our scoping review provides first guidance for regions with limited resources for surveillance, such as sub-Saharan Africa, by outlining most common associated factors and a minimum screening period of two weeks. While utilizing minimal resources effectively, this targeted surveillance could capture the majority of cases and thereby enhancing maternal patient safety.

**Systematic Review Registration:**

https://osf.io/qe7bf/

## Introduction

Surgical site infections (SSIs) in general are a major cause for post-surgical mortality and morbidity (1). With a global incidence between 3.0 and 15.0% (2), SSIs are among the most common healthcare associated infections worldwide (3). In low-resource settings, there are limited surveillance systems and hence scarce data on prevalence and associated factors of SSIs (4), but existing evidence suggests highest prevalence rates up to 30.9% in the African region (4). The most commonly performed operations around the world are caesarean sections (CSs) (5). In the African region, up to 20.0% of CSs result in SSIs leading to increased maternal morbidity and mortality, longer hospital stays and higher treatment costs (3). A recent systematic review and meta analysis on SSIs after CS reports global risk factors related to comorbidities. These reported risk factors were obesity, diabetes, hypertension, prolonged hospital stays, inappropriate timing of antibiotics, and environmental factors such as overcrowded living conditions and improper hygiene (1). In the African region, there is currently no comprehensive review on SSIs and their risk factors following caesarean section. For the African region, up to now there is no review on SSIs and associated risk factors after CS.

Our scoping review aims to synthesize literature on prevalence of SSIs after CS across sub-Saharan Africa (SSA), while elucidating associated risk and protective factors. This summarization of available evidence and hence deeper understanding of associated factors can potentially guide SSA healthcare stakeholders such as hospitals and practitioners in risk assessment and mitigation for increased maternal patient safety.

## Methods

We conducted a scoping review following the Preferred Reporting Items for Systematic Reviews and Meta-Analysis extension for Scoping Reviews (PRISMA-ScR) (6) and the Joanna Briggs Institute (JBI) methodology for scoping reviews (7). We adapted our search strategy for the African continent from the strategy used by Barth and colleagues (8) in conjunction with the definition of SSA from the World Bank (9). The protocol including the search strategy was published on Open Science Framework (OSF) (10). At the time the protocol was uploaded, no similar reviews were registered in either OSF or PROSPERO. We searched the platform OVID for publications between January 2014 and January 15, 2024 without restrictions on the language of publication or publication status. We used Rayyan (11) for deduplication and EndNote X7 (Clarivate Analytics, PA, USA) for screening and study selection (conducted by AB, RW and SM). Screening of titles and abstracts for assessment as well as screening of full text against the inclusion criteria for the review was done in pairs by the research team (AB, RW, SM, SW). Any disagreement arising at each stage of the selection process was resolved through discussion with first and supervisory authors. Studies were considered eligible if they included women who received CSs in health settings in SSA. All types of studies, including clinical trials, cohort or case-control studies were included. In the case of intervention studies, reported sample size and prevalence for the baseline were used in order to show generalisable prevalence rates. Articles in English and French were included.

Data extraction was done in double and independently by the research team (AB, RW, SM, SW) into a structured form in Microsoft Excel. The extracted variables included: author, publication year, title, journal, publication status, study type, study period, City/Country, sample size, sampling strategy, response rate, inclusion criteria, exclusion criteria, age, SSI prevalence, clinical appearance, sample collection, testing strategy, testing rate, type of infection, type of test, test name, factors investigated, factors associated, level of analysis and additional data. A forest plot was used to display results descriptively and given the hetereogeneity of included studies, a meta analysis was not performed.

## Results

### Selection of studies

**Figure 1:**
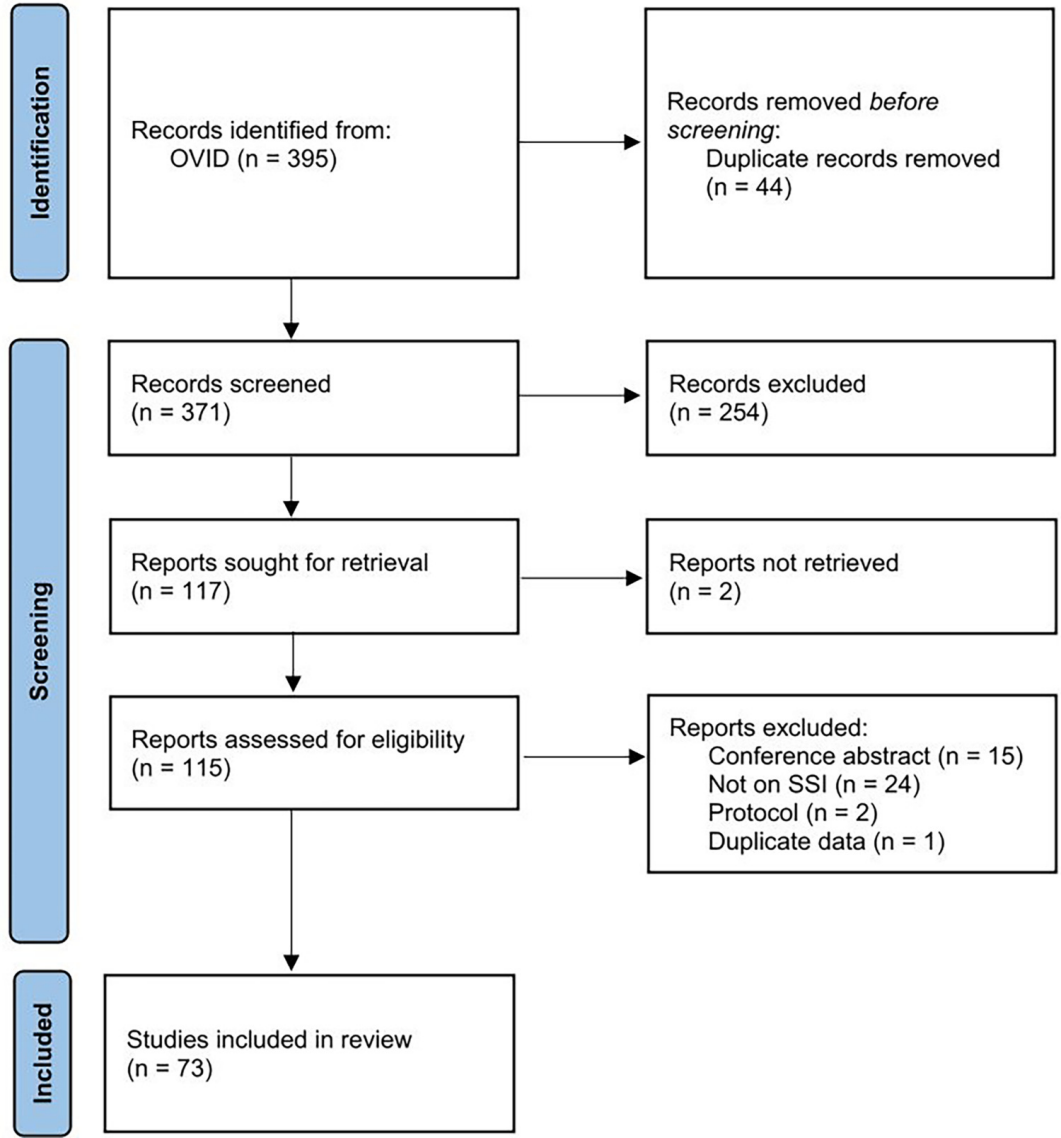
PRISMA flow diagram.

The search identified a total of 395 articles; following the removal of duplicates and critical assessment of title and abstracts, 117 potentially relevant articles were identified for full-text screening (Figure 1). Application of the pre-set eligibility criteria resulted in a final inclusion of 73 articles. All included studies were conducted between January 2009 and March 2023.

### Study characteristics

**Figure 2:**
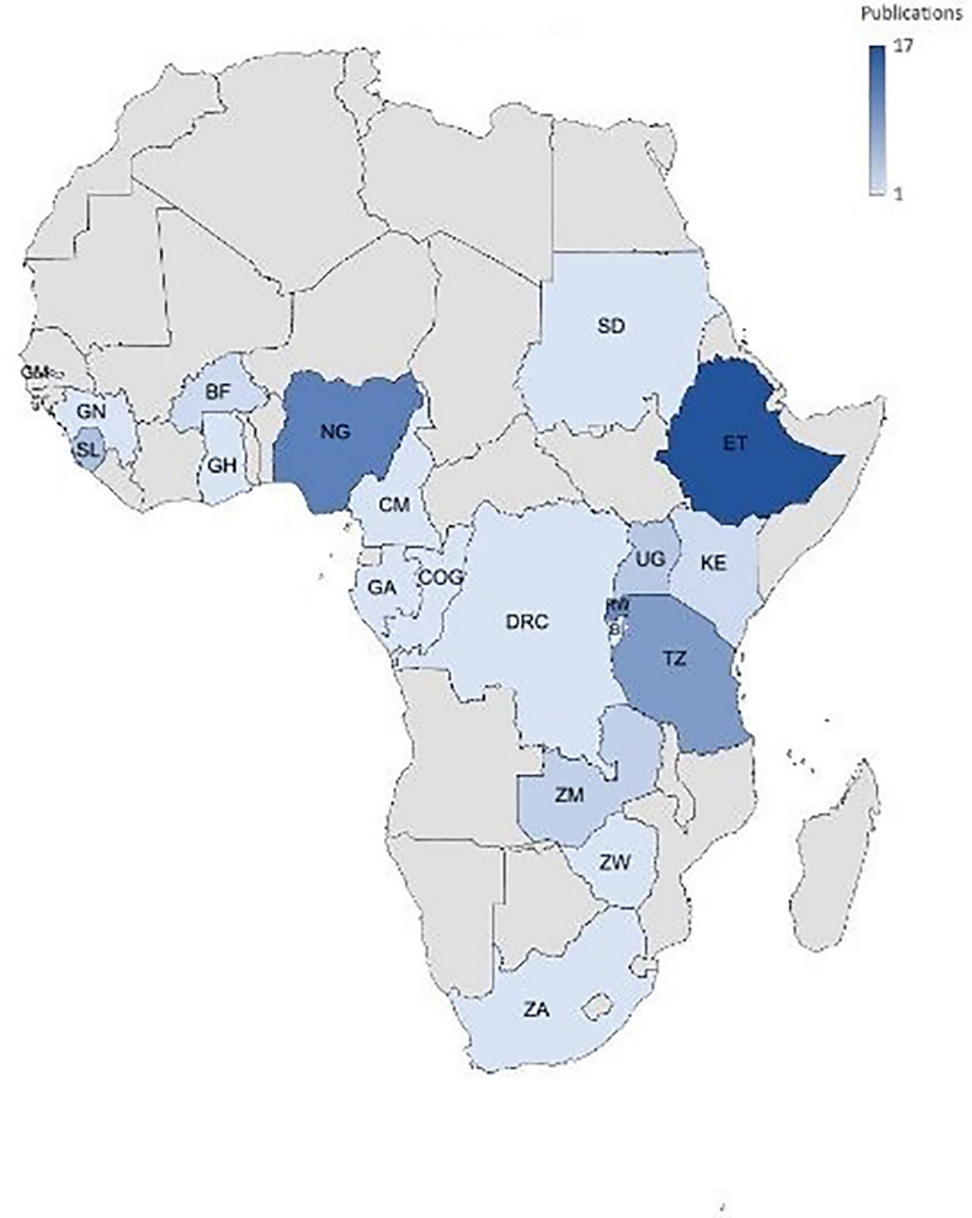
Country distribution of included publications.

The included studies contain SSI prevalence data on 51,695 women from 20 countries across SSA, with most studies from Ethiopia (17/73), Nigeria (12/73), Rwanda (11/73) and Tanzania (9/73) (Figure 2, Table 1). The most frequently analyzed health settings were university, teaching or tertiary hospitals 37.0% (27/73), followed by referral, district or regional hospitals 32.9% (24/73). No study included private hospitals. The majority of studies included women who underwent CS at the study site regardless of the indication, whereby 5.5% (4/73) focused on emergency and 2.7% (2/73) on elective CSs only (Table 1).

**Table 1:** Results of included studies.

Reference and Publication year	SSI Definition	City, country	Study Type	Sample size (*N*)	SSI prevalence % (*n*/*N*)	Follow-up (d)	Reported Appearance	Factors associated as reported (aOR, 95% CI, *p*-value)	Highest level of analysis	Bacteriological Reporting Yes/No
Adane, A. et al. (12)	CDC	Harar, Dire Dawa, Oromia, Somali, Ethiopia	Cohort	336	Overall: 7.7% (26/336)	30	8/26 before discharge	Rupture of the membrane before CS (aOR 3.75, 95% CI: 1.85–16.6)	Multivariate	Yes
					Emergency: 14.9% (17/114)		18/26 through follow-up and readmission			
					Elective: 4.1% (9/222)					
Alemye, T. et al. (13)	CDC	Harar, Ethiopia	Cross-sectional	1,069	Overall: 12.3% (131/1,069)	30	NR	General anesthesia (aOR 2.02, 95% CI: 1.34–3.02), rupture of membrane before CS (aOR 1.91, 95% CI: 1.18–3.09), post-operative hospital stay > 7d (aOR 2.24, 95% CI: 1.61–3.64), blood transfusion (aOR 4.10, 95% CI: 2.61–6.44)	Multivariate	No
					Emergency: 12.9% (105/811)					
					Elective: 10.1% (26/258)					
Ali, O. et al. (14)	Clinical Diagnosis	Gondar, Ethiopia	Cross-sectional	818	Overall: 12.2% (100/818)	NR	60/100 (60.0%) after discharge	Chorioamnionitis (aOR 6.46, 95% CI: 1.82–22.71, *p* = 0.01), Diabetes Mellitus (aOR 6.02, 95% CI: 1.69–21.36, *p* = 0.005, rupture of membrane ≥ 12 h before CS (aOR 2.94, 95% CI: 1.52–5.67, *p* = 0.001), MSAF (aOR 2.43, 95% CI: 1.23–4.81, *p* = 0.011), anemia (aOR 3.44, 95% CI: 1.56–7.56, *p* = 0.002)	Multivariate	No
					Emergency: 14.0% (96/688)					
							100/100 (100.0%) within 14 days			
					Elective: 3.1% (4/130)					
Alidina, S. et al. (15)	NR	Lake Zone, Tanzania	Case-control	Pre-intervention: 1,120 (intervention) 1,113 (control)	Pre-intervention: intervention: 6.5% (73/1,120)	30	NR	NR	NR	No
					Control: 8.1% (90/1,113)					
					Overall Pre-intervention:(163/2,233)					
				Overall pre-intervention: 2,233						
						No follow-up after discharge				
					Post-intervention: intervention: 2.3% (23/980)					
				Post-intervention: 980 (intervention)						
					Control: 2.6% (11/427)					
				427 (control)						
Aulakh, A. et al. (16)	CDC	Gambia	Case-control	682	Overall: 13.2% (90/682)	30	51/90 (58.0%) after discharge	Decision-to-incision time (*p* = 0.01), fetal status (p = 0.001), postoperative stay (*p* = 0.001), antibiotic regimen (*p* = 0.03)	Bivariate	No
					Emergency: 12.3% (70/571)					
					Elective 9.1% (3/33)					
Ayala, D. et al. (17)	CDC	Nekemte, Ethiopia	Cross-sectional	382	Overall: 8.9% (34/382)	30	NR	Age > 35 years (aOR 5.03, 95% CI: 1.69–14.95, *p* = 0.004), pregnancy-induced hypertension (aOR 5.63, 95% CI: 1.88–16.79, *p* = 0.002), prolonged Labor (>24 h) (aOR 4.12, 95% CI: 1.01–32.19, *p* = 0.048), general anesthesia (aOR 3.96, 95% CI: 1.02–15.29, *p* = 0.040), post-operative hemoglobin <11 g/dl (aOR 4.51, 95% CI: 1.84–11.07, *p* = 0.001)	Multivariate	No
					Emergency: 8.5% (28/328)					
					Elective: 11.1% (6/54)					
Azeze, G. (18)	NR	Dahir Dar, Ethiopia	Cross-sectional	383	7.8% (30/383)	30	22/30 (73.3%) after discharge	Rupture of membrane before CS (aOR 13.9, 95% CI: 2.99–64.8, *p* = 0.002), vertical skin incision (longitudinal abdominal incision) (aOR 4.77, 95% CI: 1.74–13.06, *p* = 0.001), duration of surgery >30 m (aOR 4.9, 95% CI: 1.8–13.1, *p* = 0.001), Interrupted skin closure technique (aOR 6.29, 95% CI: 2.07–19.11, *p* = 0.002)	Multivariate	No
Bizuayew, H. et al. (19)	CDC	Gojjam zone, Northwest Ethiopia	Cross-sectional	622	12.4% (77/622)	30	NR	Residence (rural) (aOR 2.30, 95% CI: 1.29–4.09, *p* = 0.005), rupture of membrane >12 h (aOR 4.61, 95% CI: 2.34–9.09, *p* = 0.001), duration of labor >24 h (aOR 3.48, 95% CI: 1.50–8.09, *p* = 0.004), hypertension (aOR 3.14, 95% CI: 1.29–7.59, *p* = 0.011), preoperative hematocrit <30% (aOR 3.22, 95% CI: 1.25–8.31, *p* = 0.016)	Multivariate	No
Brisibe, S. et al. (20)	NR	Port Harcourt, South Nigeria	Cross-sectional	Site 1	Site 1 baseline: 13.17% (54/410), follow up: 10.34% (43/416); Site 2 13.95% (42/301)	None	NR	None	Bivariate	No
				Baseline: 410, Follow up: 416 Site 2: 301						
Buambo, J. et al. (21)	NR	Brazzaville, Congo	Cross-sectional	1,063	38.4% (408/1,063)	30	NR	Age <25 years (aOR 2.0, 95% CI: 1.01–4.1, *p* = 0.04), primary education (aOR 4.1, 95% CI: 1.4–11.8, *p* = 0.09), BMI > 30 kg/m^2^ (aOR 5.9, 95% CI: 1.2–27.1, *p* = 0.02), PROM >6 h (aOR 2.2, 95% CI: 1.1–4.1, *p* = 0.02), tinted amniotic fluid (aOR 3.6, 95% CI: 1.6–7.6, *p* = 0.001), duration of surgery >45 min (aOR 21.1, 95% CI: 11.3–39.4, *p* = 0.001), no dressing (aOR 2.5, 95% CI: 1.3–4.5, *p* = 0.004), antibiotics (aOR 3.9, 95% CI: 2.2–6.8, *p* = 0.001)	Multivariate	No
Carshon-Marsh, R. et al. (22)	NR	Bo, Sierra Leone	Cohort	599	Overall: 7.5% (45/599)	30 (after discharge, telephone calls)	NR	NA	NA	No
					Mergency:7.4% (40/541)					
					Elective: 8.6% (5/58)					
Cherian, T. et al. (23)	CDC	Kirehe, Rwanda	Cross-sectional	525	9.9% (52/525)	10 ± 3, call 30	NR	NA	Multivariate	No
Chu, K. et al. (24)	CDC	Burundi, DRC, Sierra Leone	Cohort	1,276	7.3% (93/1,276)	Until discharge	Median: 6 days (range 2–17)	Age <30 years (aOR 2.1, 95% CI: 1.2–3.6, *p* = 0.013), program site (Lubutu) (aOR 0.3, 95% CI: 0.1–0.9, *p* = 0.038), PROM (aOR 2.1, 95% CI: 1.3–3.4, *p* = 0.002), neonatal death (aOR 2.7, 95% CI: 1.5–5.0, *p* = 0.001), antenatal hemorrhage (aOR 0.2, 95% CI: 0.05–1.0, *p* = 0.050)	Multivariate	No
Dayo-Dada, T. et al. (25)	NR	Ekiti State, Nigeria	Cohort	1,224	Overall: 16.0% (196/1,224)	NR	NR	Age (X²: 97.714, *p* < 0.000), Occupation (X²: 80.321, *p* < 0.000), Gravidity (X²: 175.768, *p* < 0.000), Parity (X²: 571.065, *p* < 0.000), Type of Cesarean Section (Emergency vs. Elective) (X²: 0.008, *p* < 0.000), Indication for CS (X²: 246.844, *p* < 0.000), Previous Scar (X²: 199.09, *p* < 0.000) Occupation (X²: 80.321, *p* < 0.000)	Bivariate	No
					Emergency: 16.0% (146/915)					
					Elective: 16.2% (50/309)					
De Nardo, P. et al. (26)	CDC	Dodoma, Tanzania	Cohort	467	Overall: 48.2% (225/467)	30	Median: 8 days	Senior doctor (OR 0.64, 95% CI: 0.43–0.97, *p* < 0.04), Pfannenstiel (incision) (OR 0.30, 95% CI: 0.18–0.5, *p* < 0.001), continuous intradermic (OR 0.26, 95% CI: 0.15–0.45, *p* < 0.001)	Bivariate, none significant in multivariate	Yes
					Emergency: 48.7% (207/425)					
					Elective: 43% (18/42)					
Delamou, A. et al. (27)	CDC	Multiple districts, Guinea	Cohort	7,394	Overall: 7.7% (570/7,394)	NR	NR	Year of surgery:2,014 (aOR 0.70, 95% CI: 0.57–0.84, *p* = 0.001) 2,015 (aOR 0.43, 95% CI: 0.34–0.55, *p* < 0.001) comorbidities (aOR 1.54, 95% CI: 1.25–1.90, *p* < 0.001)	Multivariate	No
					2013: 10.0% (331/3,331)					
					2014: 7.0% (138/1,971)					
					2015: 5.0% (101/2,020)					
Dessu, S. et al. (28)	CDC	Dire Dawa, Ethiopia	Cases: 119 Controls: 357	476	NA	Hospital discharge	NR	Age 20–34 years (aOR 5.4, 95% CI: 2.35–12.7), age >35 years (aOR: 8.9, 95% CI: 1.8–43.9), <4 vaginal examinations (aOR 4.2, 95% CI: 2.16–8.22), history of chorioamnionitis (aOR 5, 95% CI: 1.05–23.9), previous CS (aOR 6.2, 95% CI: 2.72–14.36), antibiotic prophylaxis (aOR 3.2, 95% CI: 1.81–5.62), perioperative haematocrit level <30% (aOR 6.9, 95% CI: 3.45–14.1), rupture of membrane >12 h (aOR 5.4, 95% CI: 1.84–15.87)	Multivariate	No
				Cases: 119						
				Controls: 357						
Di Genarro, F. et al. (29)	CDC	Freetown, Sierra Leone	Case-control	2,323	10.9% (254/2,323)	NR	Mean 4.4 ± 1.8 days	Being single (aOR 1.48, 95% CI: 1.36–1.66), abnormal BMI, low BMI (aOR 1.42, 95% CI: 1.18–1.72), high BMI (aOR 1.85, 95% CI: 1.02–2.68), admitted from home (aOR 2.35, 95% CI: 2.18–2.59), unemployed (aOR 1.74, 95% CI: 1.24–2.21), low education level (aOR 1.68, 95% CI: 1.55–1.84), presenting with PROM (aOR 1.49, 95% CI: 1.18–1.88), long decision–incision time (aOR 2.08, 95% CI: 1.74–2.24), frequent missing post CS antibiotic doses (aOR 2.52, 95% CI: 2.10–2.85), previous CS (aOR 1.27, 95% CI: 1.10–1.52)	Multivariate	No
Dlamini, L. et al.* (30)	CDC	Kampala, Uganda	Randomized Clinical Trial	432	Overall: 56.5% (244/432)	10	NR	NR	Bivariate	No
Elbur, A. et al. (31)	CDC	Khartoum, Sudan	Cross-sectional	578	8.3% (48/578)	30	NR	NR	Multivariate	No
Eleje, G. et al. (32)	NR	Ituku Ozalla/Enugu, Nigeria	Cross-sectional	Overall: 607	Overall: 6.1% (37/607)	NR	NR	None	Bivariate	No
				During COVID-19: 228	During COVID-19: 6.6% (15/228)					
				Pre-COVID-19: 379	Pre-COVID-19: 8% (22/379)					
Ernest, E. et al. (33)	CDC	Kagera/Mara regions, Tanzania	Cross-sectional	279	Baseline 13.9% (19/136)	Until discharge	NR	Implementation of the safe surgery interventions in both health care center (*p* = 0.006) and hospitals (*p* < 0.001)	Bivariate	No
					Follow-up 0.7% (1/143)					
Fletcher, R. et al. (34)	Clinical Diagnosis	Kirehe, Rwanda	Cohort	530	5.7% (30/530)	±3 (with Thermal Camera)	NR	NR	NR	No
Fletcher, R. et al. (35)	NR	Kirehe, Rwanda	Cohort	572	10.8% (62/572)	10 ± 3	NR	NR	NR	No
Gajewski, J. et al. (36)	NR	Zambia	Randomized Control Trial	1,314	1.6% (21/1,314)	NR	NR	None	Bivariate	No
Gashaw, A. et al.^#^ (37)	CDC	Hawassa, Ethiopia	Cohort	431	All Emergency: 11.8% (51/431)	NR	<7 days	Multiple vaginal examinations >5 (aOR 6.10, 95% CI: 2.15–17.35, *p* = 0.001), estimated blood loss >500 ml (aOR 3.16, 95% CI: 1.19–8.38, *p* = 0.021) duration of labor ≥12 h (aOR 4.05, 95% CI: 1.12–13.7, *p* = 0.001), rupture of membrane ≥12 h (aOR 4.12, 95% CI: 1.50–11.27, *p* = 0.006)	Multivariate	No
							22/51 (43,1%)			
							7–14 days			
							28/51 (54,9%)			
							>14 days			
							1/51 (2.0%)			
Gelaw, K. et al. (38)	Clinical Diagnosis	Maichew, Ethiopia	Cross-sectional	384	6.8% (26/384)	30	20/26 (76.9%) before discharge	Labor >24 h (aOR 3.48, 95% CI: 1.25–9.68), rupture of membrane before CS (aOR 3.68, 95% CI: 1.13–11.96) midline incision compared to Pfannestiel (aOR = 5.73, 95% CI: 2.05–16.00)	Multivariate	No
							6/26 (23.0%) after discharge			
Gentilotti, E. et al. (39)	CDC	Dodoma, Tanzania	Cohort	Total: 1,040	Overall: 30.8% (320/1,040)	30	NR	Overall/pre-intervention: Pfannenstiel incision (OR 0.29, 95% CI: 0.20–0.42, *p* < 0.001), continuous intradermic/semi-subcutaneous suture (OR 0.32, 95% CI: 0.23–0.46, *p* < 0.001); Pre-intervention: higher experience of the surgeon (OR 0.64, 95% CI: 0.43–0.97, *p* = 0.038)	Multivariate	Yes
					Emergency: 31% (299/964)					
				Pre: 467	Elective: 27.6% (21/76)					
					Pre-Intervention: 48.2% (225/467)					
				Post: 573						
					Post-Intervention: 16.6% (95/573)					
								Post-intervention: younger age (OR 2.38, 95% CI: 1.38–4.09, *p* = 0.001), absorbable stiches (OR 0.47, 95% CI: 0.27–0.81, *p* = 0.006)		
								multivariate: lack of pre-incision antibiotic prophylaxis (OR 3.59, 95% CI: 1.92–6.70, *p* < 0.001), skin disinfection with Dettol/Ethanol (OR 2.40, 95% CI: 1.00–5.74, *p* = 0.050), absorbable suture (OR 0.52, 95% CI: 0.28–0.97, *p* = 0.040), normal BMI (18,5–24,9) (OR 0.63, 95% CI: 0.40–0.99, *p* = 0.045)		
Gidiri, M./Ziruma, A. (40)	NR	Partirenyatwa/Harare, Zimbabwe	Randomized Control Trial	232	4.7% (11/232)	42	NR	None	Bivariate	No
Hedt-Gauthier, B. et al. (41)	Clinical Diagnosis	Kirehe District, Rwanda	Cohort	569	10.7% (61/569)	10	NR	NR	NR	No
Igwemadu, G. et al. (42)	NR	Keffi, Nigeria	Randomized Control Trial	162	7.0% (11/162)	14 after discharge	NR	None	Bivariate	No
Kabore, B. et al. (43)	NR	Fada N'Gourma/Diapage, Burkina Faso	Case-control	198: cases: 99, controls: 99	NR	NR	NR	Hyperthermia upon admittance (aOR 2.37, 95% CI: 1.9–5.3, *p* = 0.035), caput succedaneum (aOR 7.0, 95% CI: 2.5–16.7, *p* = 0.001) difficult extraction of the fetus (aOR 3.69, 95% CI: 1.26–6.3, *p* = 0.02)	Multivariate	No
Kasanga, M. et al. (44)	NR	Lusaka, Zambia	Cross-sectional	838	6.0% (50/838)	NR	NR	Secondary education (OR 0.38, 95% CI: 0.15–0.95, *p* < 0.038), emergency CS (OR 6.25, 95% CI: 2.83–13.80, *p* < 0.001), oral antibiotics post CS (OR 0.22, 95% CI: 0.05–0.96, *p* < 0.045), performing facility (OR 0.06, 95% CI: 0.02–0.17, *p* < 0.001), 8–15d IV antibiotic treatment (OR 18.04, 95% CI: 6.61–49.28, *p* < 0.001),	Bivariate	No
Kateera, F. et al. (45)	NR	Kirehe, Rwanda	Randomized Control Trial	871	10.9% (95/871)	30	NR	None	Bivariate	No
Ketema, D. et al. (46)	CDC	Amhara, Ethiopia	Cohort	520	Overall: 25.4% (132/520)	30	Median: 8 days	Not able to read and write (AHR 1.30, 95% CI: 1.19–2.11), no antenatal care (AHR 2.16, 95% CI: 1.05–4.53), previous CS (AHR 1.21, 95% CI: 1.11–2.31), HIV positive (AHR 1.39, 95% CI: 1.21–2.57), emergency procedure (AHR 1.13, 95% CI: 1.11–2.43), vertical skin incision (AHR 2.60, 95% CI: 1.05–6.44), rupture of membrane (AHR 1.50, 95% CI: 1.31–1.64), multiple vaginal examination (AHR 1.88, 95% CI: 1.71–3.20)	Multivariate	No
					Emergency: 26.3% (111/422)					
					Elective: 21.4% (21/98)					
							(IQR 5–13)			
Kpagoi, S. et al. (47)	WHO protocol for surgical site infection	Bo, Sierra Leone	Cross-sectional	596	2.5% (15/596)	30	NR	NR	NR	No
Lijaemiro, H. et al. (48)	CDC	Addis Ababa, Ethiopia	Cohort	166	15.1% (25/166)	30	1–10 days (9.4%)	One-year increment in age (aOR 1.50, 95% CI: 1.17–1.93, *p* < 0.001), gestational age (aOR 0.02, 95% CI: 0.00–0.29, *p* < 0.004), one-minute increment of duration of surgery (aOR 1.12, 95% CI: 1.03–1.20, *p* < 0.009), ≥5 vaginal examinations (aOR 13.08, 95% CI: 1.02–168.00, *p* < 0.048), CS at term (aOR 0.02, 95% CI: 0.00–0.29)	Multivariate	No
							11–17 days (15.6%)			
							25–30 days (1.4%)			
Lukabwe, H. et al.* (49)	NR	Mbarara, Uganda	Randomized Control Trial	96	30.2% (29/96)	30	NR	Pre-operative baths with Chloroxylenol (adjusted RR 0.1, 95% CI: 0.03–0.33, *p* < 0.001)	Multivariate	No
Mezemir, R. et al. (50)	CDC	Addis Abeba, Ethiopia	Cohort	741	11.6% (86/741)	30	Mean: 9 days	2–3 antenatal care visits (aOR 3.11, 95% CI: 1.69–5.75), delayed antenatal booking (aOR 6.99, 95% CI: 2.09–23.32), PROM (aOR: 2.10, 95% CI: 1.0–4.24), multiple vaginal examinations (aOR 4.21, 95% CI: 1.35–6.92), public hospitals (aOR 11.1, 95% CI: 1.48–45.14), hospital stay <7 days (aOR 0.37, 95% CI: 0.15–0.91), transversal incisions (aOR 0.38, 95% CI: 0.15–0.91)	Multivariate	Yes
							(Range 8–10)			
Mivumbi, V. et al. (51)	NR	Kigali, Rwanda	Randomized Control Trial	132	3.8% (5/132)	14	All within 14 days (14 days was the study follow-up time)	NR	NR	Yes
Miyoshi, Y. et al. (52)*	NR	Zimba, Zambia	Cohort	266	2.3% (6/266)	NR	NR	None	Bivariate	No
Mohammed, S. et al. (53)	CDC	Kano, Nigeria	Randomized Control Trial	154	Overall: 8.4% (13/154)	30	Mean: 7.9 ± 3.8 days	None	Bivariate	No
					Emergency: 17.0% (8/47)					
					Elective: 4.7% (5/107)		NR			
Molla, M. et al. ^#^ (54)	CDC	Debretabor, Ethiopia	Cross-sectional	334	8.1% 27/334)	30	Before discharge: 3/27 (11.1%)	Pregnancy induced hypertension (aOR 4.75, 95% CI: 1.62–13.92), chorioamnionitis (aOR 4.37, 95% CI: 1.53–12.50), midline skin incision (aOR 5.19, 95% CI: 1.87–14.37), post-operative hemoglobin l < 11 g/dc (aOR 5.28, 95% CI: 1.97–14.18)	Multivariate	No
							Post Discharge: 24/27 (88.9%)			
Mothiba, M. et al. ^†^ (55)	CDC	Pretoria, South Africa	Randomized Control Trial	207	0 (0/207)	30	NR	None	Bivariate	No
Mpogoro, F. et al. ^#^ (56)	CDC	Mwanza, Tanzania	Cohort	345	10.9% (34/312)	30	Median: 7 days	Hypertensive disorder (HR 2.9, 95% CI: 1.4–6.4, *p* < 0.006); contaminated wound (HR 2.5, 95% CI: 1.2–5.1, *p* < 0.016), multiple vaginal examinations (HR 2.6, 95% CI: 1.3–5.3, *p* < 0.008), operation done by intern doctor (HR 4.2,95% CI: 1.8–9.5, *p* < 0.001), severe anemia (HR 3.8, 95% CI: 1.2–12.4, *p* < 0.028), duration of procedure >1 h (HR 2.3, 95% CI: 1.1–4.8, *p* < 0.030)	Multivariate	Yes
							(IQR: 6–9)			
Mukantwari, J. et al. (57)	CDC	Kirehe, Rwanda	Cohort	671	10.7% (72/671)	30	11 days: 33/671 (4.9%)	Having health insurance (aOR 0.06, 95% CI: 0.01–0.58, *p* < 0.013), higher economic status (aOR 2.88, 95% CI: 1.39–5.97, *p* < 0.004)	Multivariate	No
							30 days: 39/671 (5.8%)			
Ketemaw, N./ Dereje Zeleke, B. (58)	CDC	Kaffa Zone, Ethiopia	Cohort	368	10.3% (38/368)	NR	NR	None	Bivariate	No
Ngonzi, J. et al. (59)	NR	Mbarara, Uganda	Cohort	678	Pre-intervention: 14.5% (29/200)	Only pre-discharge follow-up	NR	NR	Bivariate	No
					During intervention: 7.4% (17/230)					
					Post-intervention: 10.5% (26/248)					
Ngowa, J. et al. (60)	NR	Yaoundé, Cameroun	Cohort	460	1.7% (8/460)	30	NR	NR	Bivariate	No
Nguhuni, B. et al. (61)	CDC	Dodoma, Tanzania	Cohort	374	12.0% (45/374)	30	Median: 8 days	None	NR	No
							(IQR: 7–11)			
Njoku, C. et al. (62)	CDC	Calabar, Nigeria	Cohort	600	Overall: 8.5% (51/600)	30	NR	Emergency CS (aOR 4.71, 95% CI: 3.19–5.35, *p* < 0.001), indication for CS (aOR 1.35, 95% CI: 1.00–1.65, *p* < 0.002), duration of membrane rupture (aOR 0.52, 95% CI: 0.32–0.95, *p* < 0.002), duration of labor (aOR 0.47, 95% CI: 0.20–0.79, *p* < 0.001), intra-operative blood loss >1 L (aOR 1.22, 95% CI: 1.17–2.90, *p* < 0.048), duration of surgery <1 h (aOR 0.03, 95% CI: 0.01–0.07, *p* < 0.028), post-operative packed cell volume <30% (aOR 2.60, 95% CI: 1.46–4.12, *p* < 0.002)	Multivariate	Yes
					Emergency: 11.0% (45/410)					
					Elective: 3.2% (6/190)					
Nkurunziza, T. et al. (63)	NICE	Kirehe, Rwanda	Cohort	550	10.9% (60/550)	10 ± 3	All within 10 days (10 days was the study follow-up time)	Transport cost >1.1 EUR (aOR 2.42, 95% CI: 1.31–4.49, *p* < 0.005), housewife (aOR 2.93, 95% CI: 1.08–7.97, *p* < 0.035), Skin preparation with one antiseptic (aOR 4.42, 95% CI: 1.05–18.57, *p* < 0.043)	Multivariate	No
Nkurunziza, T. et al. (64)	CDC	Kirehe, Rwanda	Cohort	787	POD 10 ± 3: 4.2% (30/715)	Home visit with telemedicine: 10 ± 3	All within 10 days (10 days was the study follow-up time)	NR	NR	No
					POD 11 ± 3: 5.4% (38/707)					
						Hospital visit: 11 ± 3,				
Odada, D. et al. (65)	NHSN	Nairobi, Kenya	Case-control	Total: 1,262	Overall: 2.1% (27/1,262)	30	Out of the 27 SSI (4 dropped due to missing information)	None	Bivariate	Yes
					Out of 69 study participants: Emergency: 35.3% (12/34)					
				(69 study participants)			14 days: 13/23 (56.0%)			
					Elective: 31.4% (11/35)					
							15–30 days: 10/23 (43.5%)			
Ogah, C. et al. (66)*	NR	Abakaliki, Nigeria	Randomized Control trial	302 (152 control, 150 intervention)	Overall: 7.3% (22/302)	30	Mean: 4.1 days	None	Bivariate	Yes
					Intervention: wound infection: 5.3% (8/150)					
					Control: wound infection: 9.2% (14/152)					
Onuzo, C. et al. (67)	CDC	Accra, Ghana	Cohort	474	Overall: 12.9% (61/474)	30	Before discharge from hospital: 13/61 (21.3%)	Being single (aOR 4.81, 95% CI: 1.21–19.17, *p* < 0.03), alcohol consumption >3l/week (aOR 5.97, 95% CI: 1.32–26.98, *p* < 0.02), duration of labor ≥ 8 h (aOR 75.67, 95% CI: 6.61–866.24, *p* < 0.01), emergency CS (aOR 4.66, 95% CI: 1.22–17.75, *p* < 0.02), stored water used for pre-operative scrub rather than running water (aOR 18.60, 95% CI: 3.55–97.56, *p* < 0.01), vertical midline skin incision (aOR 12.55, 95% CI: 2.14—73.63, *p* < 0.05)	Multivariate	Yes
					Emergency: 16.5% (51/309)					
							Detected in post-discharge follow-up: 48/61 (78.8%)			
					Elective: 6.1% (10/165)					
							Median: 7 days			
Onyegbule, O. et al. (68)	NR	Nnewi, Nigeria	Cross-sectional	120	Overall: 12.5% (15/120)	Follow-up and diagnosis done at day 4, additional follow-up not NR	Follow-up and diagnosis done at day 4, additional follow-up not NR	For emergency CS: rupture of membrane <24 h (aOR 0.11, 95% CI: 0.03–0.47, *p* < 0.003), duration of labor <12 h (aOR 0.07, 95% CI: 0.01–0.32, *p* < 0.001), Pfannenstiel incision (aOR 0.21, 95% CI: 0.05–0.91, *p* < 0.038)	Multivariate	No
					Emergency: 20.0% (12/60)					
					Elective: 5.0% (3/60)					
Oyeyem, N. et al. ^†^ (69)	NR	Lagos, Nigeria	Randomized Control Trial	190	21.6% (41/190)	14 days and 6 weeks	NR	None	Bivariate	No
Peter, E./Ali Seif, S. (70)	NR	Dodoma, Tanzania	Cross-sectional	183	20.8% (38/183)	14	NR	Poor wound care (aOR 5.95, 95% CI:1.76–20.17, *p* < 0.004), earth/sand houses (aOR 4.32, 95% CI: 1.11–16.83, *p* < 0.03)	Multivariate	Yes
Rabiu, K. et al. (71)	NR	Lagos, Nigeria	Cohort	906	Overall: 19.4% (176/906)	Until Discharge	NR	Preoperative anemia (aOR 1.88, 95% CI: 1.03–3.41, *p* < 0.040, diabetes mellitus (aOR 7.94, 95% CI: 1.60–39.27, *p* < 0.011), HIV infection (aOR 6.34, 95% CI: 1.74–23.06, *p* < 0.005), prolonged operation time (aOR 2.30, 95% CI: 1.19–4.42, *p* < 0.013), excessive blood loss at surgery (aOR 5.05, 95% CI: 2.18–11.66, *p* < 0.000), chorioamnionitis (aOR 9.00, 95% CI: 1.37–59.32, *p* < 0.022)	Multivariate	No
					Emergency: 24.1% (143/594)					
					Elective: 10.6% (33/312)					
Robb, K. et al. (72)	screening protocol	Kirehe, Rwanda	Cross-sectional	173	9.8% (17/173)	30	NR	Lack of water (OR 2.6, *p* < 0.027)	Bivariate	No
Sawadogo, Y. et al. (73)*	Clinical Diagnosis	Ouagadougou, Burkina Faso	Cross-sectional	1,998	3.5% (70/1,998)	NR	Mean: 6,7 ± 2.3 days	None	NA	Yes
Scherbaum, M. et al. (74)	RKI/CDC	Lambaréné, Gabon	Cohort	80	6.3% (5/80)	Until discharge	All detected before discharge	NR	NR	No
Sway, A. et al. (75)	CDC	Kiambu, Kenya	Cohort	600	6.7% (40/600)	30	NR	Administration of pre-operative antibiotic prophylaxis (OR 0.41, 95% CI: 0.20–0.82, *p* < 0.01)	Bivariate	No
Ugadu, I. et al. (76)	CDC	Abakaliki, Nigeria	Randomized Control Trial	239	4.6% (11/239)	14	NR	Preoperative cleansing on maternal infectious morbidity (RR 0.13, 95% CI: 0.05–0.36, *p* < 0.000)	Bivariate	No
Velin, L. et al. (77)	CDC	Kirehe, Rwanda	Cohort	795	5.7% (45/795)	11 ± 3	Range: 8—14 days	NR	NR	Yes
Waalewijn, B. et al. (78)	NR	Sierra Leone	Cohort	1,174	3.7% (36/984)	NR	NR	NR	Bivariate	No
Wae, M. et al. (79)	NR	Arba Minch, Ethiopia	Cohort	416	12.0% (50/416)	NR	NR	NR	NR	No
Wendmagegn, T. et al. (80)	CDC	Mekelle, Ethiopia	Cohort	206	Overall: 11.7% (24/206)	NR	NR	PROM (aOR 8.82, 95% CI: 21.71–35.82, *p* < 0.002), prolonged labor (aOR 16.17, 95% CI: 2.85–91.82), *p* < 0.006), rural setting (aOR 5.67, 95% CI: 1.57–20.48), HIV positive (aOR 6.98, 95% CI: 1.38–35.27, *p* < 0.019), chorioamnionitis (aOR 16.17, 95% CI: 2.85–91.82, *p* < 0.002), blood loss <1000 ml (aOR 0.01 95% CI: 0.02–0.57, *p* < 0.01)	Multivariate	No
					Emergency: 12.4% (24/193)					
					Elective: 0.0% (0/13)					
Westen, E. et al. (81)	NR	Lindi/ Masasi, Tanzania	Randomized Control Trial	181	8.3% (15/181)	30	NR	NR	NR	No
Wodajo, S. et al. (82)	CDC	Hawassa Town, Ethiopia	Cross-sectional	592	11.0% (65/592)	NR	Before discharge: 64/65 (98.4%)	Prolonged labor >24 h (aOR 6.78, 95% CI: 2.54–18.00), PROM < 12 h (aOR 5.83, 95% CI: 2.14–15.89), 1–4 digital vaginal examinations (aOR 2.91, 95% CI: 1.21–6.99), 5 digital examinations (aOR 8.59, 95% CI: 1.74–42.23), duration of surgery >1 h (aOR 12.32, 95% CI: 5.46–27.77), wound contamination class III (aOR 9.61, 95% CI: 1.84–50.06), conducted by junior professionals (GP) (aOR 7.06, 95% CI: 1.62–30.70) MSc students (aOR 8.31, 95% CI:1.79–28.52), postoperative hemoglobin <11 mg/dl (aOR 2.62, 95% CI: 1.21–5.69)	Multivariate	No
Woodd, S. et al. (83)	CDC	Dar es Salaam, Tanzania	Cohort	146	8.2% (12/146)	7telephone interview	NR	NR	Multivariate	No
Zubairu, U. et al. (84)	CDC	Zaria, Nigeria	Randomized Control Trial	170	11.2% (19/170)	14	Mean: 10.2 ± 3.6 days	Level of education (tertiary) (aOR 0.24, 95% CI: 0.08–0.75, *p* < 0.014), parity <4 (aOR 0.23, 95% CI: 0.08–0.67, *p* < 0.007), no endometritis (aOR 0.10, 95% CI: 0.03–0.29, *p* < 0.000), no febrile morbidity (aOR 0.12, 95% CI: 0.02–0.72, *p* < 0.020)	Multivariate	No
					Emergency: 13.9% (14/101)					
					Elective: 7.2% (5/69)					

CDC, center for disease control and prevention; CI, confidence interval; AHR, adjusted hazard ratio; aOR, adjusted odds ratio; BMI, body mass index; CD, caesarean delivery; CS, c-section; GP, general practitioner; HIV, human immunodeficiency virus; HR, hazard ratio; Md, median; IQR, interquartile range; MSAF, meconium-stained amniotic fluid; MSc, master of science; NA, not applicable; NHSN, national healthcare safety work; NICE, national institute for health and care excellence; NR, not reported; PCV, pac,ed cell volume; POD, post-operative day; PROM, premature rupture of membrane; RKI, Robert-Koch-Institute; RR, relative risk; SD, standard deviation; SSI, surgical site infection; WHO, world health organization.

* This study only included emergency C-sections.

^#^ Studies that had both elective/emergency C-sections included, whereby SSIs only appeared in those who had an emergency C-section.

^†^ Studies which only had elective C-sections included.

In terms of study type, 43.8% (32/73) of included studies were cohort, 28.8% (21/73) cross-sectional studies and 19.2% (14/73) randomized control studies and 8.2% (6/73) case control (Table 1).

Regarding the applied SSI definition, the majority of studies (54.5%, 40/74) reported to have used the Center for Disease Control guidelines (85), but only a slim majority of 52.5% (21/40) of these also conducted patient follow-up for the full recommended 30-day period. In total, a 37.0% (27/73) minority of studies reported full tracking of patients up to 30 days regardless of the definition applied (Table 1).

### SSI rate and appearance time

**Figure 3:**
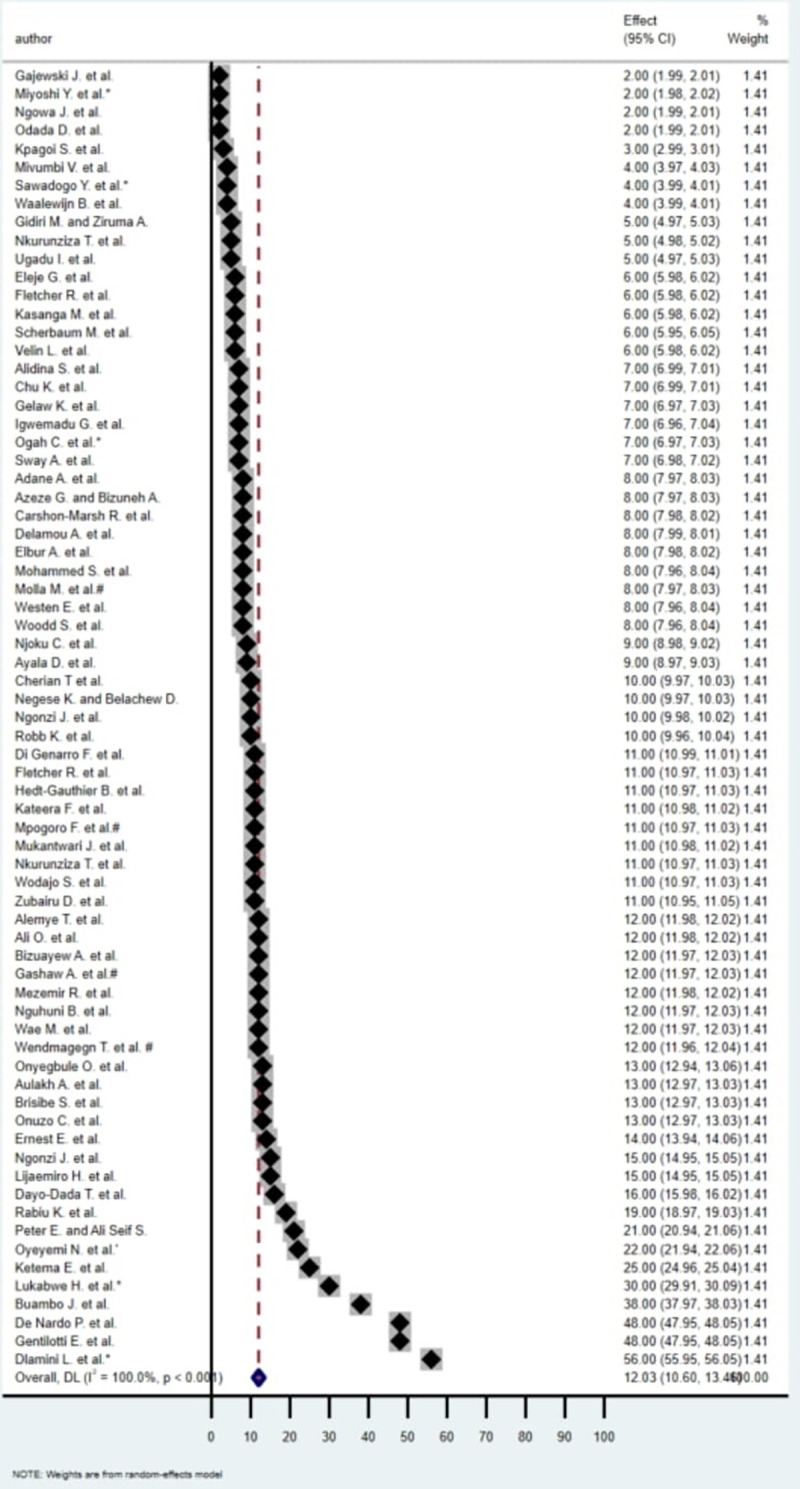
Forest plot of SSI rates of included publications.

Reported SSI rates among studies ranged from 2.0% (36)–56.0% (30) (Figure 3). The forest plot showed that the studies were highly heterogeneous (*I*^2^ = 100.0%, *p* < 0.001), and only 11.0% (8/73) of studies showed an SSI rate above 20.0% (Table 1). Regarding the indication for CSs, SSI rates for emergency CS ranged between 7.4% (22) and 48.7% (26), whereby SSI after elective CS from 3.1% (14)–43.0% (26).

Superficial SSIs had rates as high as 100.0% and were described in 32.3% (24/73) of studies followed by 24.7% (18/73) of studies reporting deep SSIs with highest rate of 32%, and 16.4% (12/73) detailing organ SSIs with rates up to 30.9% (Table 2).

**Table 2:** SSI classification in included studies.

Publication	SSI Classification (superficial/cutaneous/incisional, deep, and organ, other)
Adane, A. et al. (12)	Superficial: 19/26 (73.1%)
	Deep: 3/26 (11.5%)
	Organ: 4/26 (15.38%)
Ali. O. et al. (14)	Superficial: 88/100 (88.0%)
	Deep: 12/100 (12.0%)
	Organ: NA
Aulukah, A. et al. (16)	Superficial: 88/89 (98.9%)
	Deep: NA
	Organ: 1/89 (1.1%)
Azeze, G./Bizuneh, A. (18)	Superficial: 23 (76.7%)
	Deep: 7 (23.3%)
	Organ: NA
Brisibe, S. F. A. et al. (20)	**Site 1**
	Superficial: 16/54 (29.6%)
	Deep: 25/54 (46.3%)
	Organ: 13/54 (24.1%)
	**Site 2**
	Superficial: 10/42 (23.8%)
	Deep: 19/42 (45.2%)
	Organ: 13/42 (31.0%)
Buambo, J. R. G. et al. (21)	Superficial: 90/408 (22.0%)
	Other: Endometritis 163/408 (40.0%)
	Pelviperitonitis: 65/408 (16.0%)
Chu, K. et al. (24)	Superficial: 85/93 (91.4%)
	Deep: 7/93 (7.5%)
	Unknown: 1/93 (1.1%)
De Nardo,P. et al. (26)	Superficial: 138/225 (61.4%)
	Deep: 69/225 (30.6%)
	Organ/spaces: 5/225 (2.3%)
	Unknown: 13/225 (5.7%)
Delamou, A. et al. (27)	Cutaneous:
	2013: 88.0%
	2014: 93.0%
	2015: 89.0%
Di Genarro, F. et al. (29)	Superficial: 90/254 (35.4%)
	Deep: 98/254 (38.6%)
	Organ/Space: 66/254 (26.0%)
Gashaw A, et al. (37)	Superficial: 33/51 (64.7%)
	Deep: 15/51 (29.4%)
	Organ/space: 3/51 (5.9%)
Gentilotti, E. et al. (39)	**Pre:**
	Superficial: 138/225 (61.3%)
	Deep/involving organ and/or space: 74/225 (32.9%)
	**Post:**
	Superficial: 80/95 (84.2%)
	Deep/involving organ and/or spaces: 11/95 (11.6%)
	**Overall:**
	Superficial: 218/320 (68.1%)
	Deep/Organ: 85/320 (26.6%)
Lijaemiro et al. (48)	Superficial: 17/25 (68.0%)
	Deep: 8/25 (32.0%)
Mezemir, R. et al. (50)	Superficial: 81/86 (94.1%)
Molla, M. et al. (54)	Superficial incisional: 7/27 (27.1%)
Mpogoro, F. et al. (56)	Superficial: 21/34 (61.8%)
	Organ space: 8/34 (23.5%)
	Deep: 5/34 (14.7%)
Nguhuni, B. et al. (61)	Superficial: 42/45 (93.3%)
	Deep: 2/45 (4.4%)
	Oragn/space: 1/45 (2.2%)
Nkurunziza, T. et al. (63)	Superficial: 45/60 (75.0%)
Odada, D. et al. (65)	Superficial: 18/23 (78.3%)
	Deep: 5/23 (21.7%)
Onuzo, C. et al. (67)	Superficial: 41/61 (67,2%)
	Deep incisional: 18/61 (29.5%)
	Organ space: 2/61 (3.3%)
Rabiu, K. A.et al. (71)	Superficial: 139/176 (79.0%)
	Deep: 37/176 (21.0%)
Sway, A. et al. (75)	**Thika:**
	Superficial: 11/12 (91.7)
	Organ/space: 1/12 (8.3)
	**Kiambu:**
	Superficial: 18/28 (64.3%)
	Deep: 7/28 (25.0%)
	Organ/Space: 3/28 (10.7%)
	Unknown: 1/28 (3.6%)
Velin, L. et al. (77)	Superficial: 40/45 (88.9%)
	Deep: 5/45 (11.1%)
Wodajo, S. et al. (82)	Superficial: 46/65 (70.8%)
	Deep: 17/65 (26.1%)
	Organ: 2/65 (2.3%)

Appearance time of an SSI was reported by 42.5% (31/73) of included studies. A majority 61.3% (19/31) of those studies stated that most SSIs appear during the first two weeks after CS. Additionally, some studies reported SSI appearance time terms of pre/post-discharge (8/31) out of which a 75% majority (6/8) of SSIs appeared during post-discharge (Table 1).

### Associated factors

Associated factors in the development of SSIs were reported in 38.0% (28/73) of the studies (Table 1). Duration of labour ≥8 h presented as the strongest risk factor (aOR 75.6) (67) and was mentioned in 9.6% (7/73) of the included studies (Table 1). Extended surgical duration, mentioned six times as a risk factor, also presented a substantial risk (aOR 21.1) (21) (Table 1).

A high number (greater than five) of vaginal examinations was mentioned multiple times with a risk of up to 13.1 (48), as well as stored water with aOR of 18.6 (48).

Additionally, chorioamnionitis (aOR 16.2) (80), an infection of the amniotic fluid, also significantly elevated the risk of post-caesarean SSI and was mentioned in five of the included studies (Table 1).

Premature rupture of membrane (PROM), was observed in 14 of the included studies (Table 1), making it the most frequent risk factor with reported aOR of up to 13.9 (18). Anaemia (also reported as low-haemoglobin/haematocrit/packed cell volume) throughout the surgical intervention was mentioned nine times (Table 1) with the highest aOR of 6.9 (28). Vertical/midline skin incisions (aOR 12.6) (67) were also notably high-risk factors compared to Pfannenstiel/transversal incisions which were reported as protective factors (aOR 0.21) (68).

The analysis of protective factors for post-CS outcomes identified several key factors, whereby CS at term (aOR 0.02) (48) presented the strongest protective effect against SSIs.

Having health insurance (aOR 0.06) (57) as well as tertiary level of education (aOR 0.24) (84), demonstrated as protective factors. Within the same study, all other associated factors were found to be protective including parity <4 (aOR 0.23) (84). Women with a normal body mass index (18.5–24.9) showed a protective aOR of 0.63 (39).

As opposed to the demonstrated risk factors of prolonged duration of labour, extended surgery duration and PROM, a <12 h duration of labour (aOR 0.07) (68), less than a 1 h surgical intervention (aOR 0.03) (62) and <24 h of membrane rupture (aOR 0.52) (62) were reported as protective factors.

Other surgical factors such as intraoperative blood loss of <1,000 ml (aOR 0.10) (80) and absorbable sutures (aOR 0.52) (39) were strong protective factors. Lastly, hospital stays of less than seven days had a protective aOR of 0.37 (50).

### Bacteriological profiles

Only a minority of studies (17.8%; 13/73) reported bacteriological test results (Table 1). *S*taphylococcus *aureus* (*S. aureus*) was isolated in all thirteen of the studies with detection rates of up to 52.6% (70), and three of these studies also reported Methicillin-resistant *S. aureus* (MRSA) (26, 39, 67) which in turn was detected in rates of up to 79.0% (26, 39). One study measuring a 79.0% MRSA rate was able to reduce to a rate of only 21.0% following interventive measures (39).

Gram-negative bacteria were also prominent, particularly *Escherichia coli* which was identified in nine of the studies, and *Klebsiella pneumoniae* which was mentioned in seven studies, with two other studies also reporting *Klebsiella species*. Gram-negative bacteria was also reported as being resistant to ampicillin (100.0%), amoxicillin/clavulanate (93.0%), and trimethoprim/sulfamethoxazole (78.5%) (56) and susceptible or resistant to ceftriaxone (92.1%) and cefepime (84.6%) (77).

## Discussion

This scoping review has a wide geographical representation with included data from 20 SSA countries, whereby most studies came from Ethiopia and Nigeria, possibly reflecting these countries as research epicenters in the region.

Our scoping review found a varying rate of SSIs. However, a large majority reported rates equal or below 20%, reflecting the WHO reporting for the African region (3).

CS are the most commonly performed major operation globally (5), thus surveillance of SSI after CS can be a good starting point for SSI surveillance (3). However, full patient follow-up to day 30 was only conducted in a small minority of studies, demonstrating a possible discordance between international guidelines and their feasibility in resource-limited settings. Given that the majority of SSI were diagnosed within the first two weeks after CS, this time frame could potentially be taken into consideration in the pending update to the WHO SSI surveillance protocol for resource-limited settings (3). Additionally, eight studies examined SSI occurrences in relation to discharge timing, with six reporting that SSIs primarily emerged post-discharge. This highlights the need to follow-up discharged patients, especially within the first two weeks after CS.

Given the limited-resource setting in SSA, the introduction of surveillance systems could start by targeted SSI screening of patients with the risk factors identified as most frequent such as PROM, prolonged labor, duration of surgery, anemia and multiple vaginal examinations. In addition to this targeted surveillance, we would recommend considering measures to mitigate certain risk factors, such as, treating anemia, applying hygiene measures during vaginal examinations and ensuring the provision of clean water. Additionally, certain surgical techniques such as Pfannenstiel (horizontal/transversal) incisions and absorbable sutures should be prioritized given their potential to minimize foreign body reaction (86) and decrease the likelihood of SSIs. SSIs can lead to increased hospital stays, costs, morbidities and mortalities, making their prevention and prompt management a priority (87). Incorporating these protective factors into clinical practice can potentially enhance patient recovery and reduce complication rates and hospitalisation duration. Such incorporation should be done in accordance with the WHO global guidelines for SSI prevention which also specify known protective measures (88).

Our scoping review found a considerable lack in the provision of data on bacteriological profiles. This weakness is in line with recent literature, showing that only 1.0% of laboratories in SSA are formally assigned to deliver bacterial testing (36). However, our synthesized data highlights *S*taphylococcus *aureus* as the most frequently reported pathogen causing SSI aligning with data from a recent meta-analysis (30). This is an area of concern considering corresponding reported rates of Methicillin-resistant *S. aureus*. Prevalence of Gram-negative *Klebsiella pneumonia* and *Escherichia coli* also emphasizes the burden of enterobacteria in SSI.

Current literature promotes antibiotic stewardship measures such as selecting the proper antibiotic for prophylaxis in accordance with current resistance data, but acknowledges that stewardship recommendations can be difficult to implement in settings like SSA that have limited antibiotic resources and resistance data (89). These findings, therefore, highlight the urgent need for enhanced bacteriological surveillance and antimicrobial resistance monitoring to inform effective SSI management strategies in the SSA region.

### Limitations

This scoping review has several limitations. Despite our inclusive approach and the inclusion of all studies conducted in SSA, only 20 out of 48 of SSA countries were represented with highest representation of Ethiopia and Nigeria. We did not limit our search to English articles, nevertheless, we were only able to retrieve two French publications. As such, we reran the OVID search using French terms, but still did not retrieve additional French articles. Our search strategy only included studies published from 2014 onwards, potentially excluding older but relevant data. Due to the lack of available data in included studies, as well as missing correlation between symptom data and SSI, and vague distinction between wound infection symptoms and other issues such as endometritis, we dropped the analysis of SSI symptom data. Lastly, no private hospitals were included in the selected studies, therefore our findings may not be applicable in those settings.

## Conclusion

Findings from this study can aid those who wish to follow the WHO recommendations in using post-caesarean section SSIs as a practical entry point for healthcare associated infection surveillance. However, low reporting on aspects such as full 30-day follow-up and bacteriological testing from included studies suggests difficulty in implementation of some surveillance measures. As most reported SSIs surfaced within the first two-weeks, this time frame can be taken into consideration as a first step in surveillance implementation. Regions such as SSA that have limited-resources for surveillance and treatment can also consider targeted SSI screening of patients with frequent risk factors, and promotion of reported protective measures. Furthermore, bacteriological diagnostic capacity building is greatly needed in the region in order to improve data gaps and antibiotic treatment recommendations. Utilization of these recommendations can ideally contribute towards improved safety for women undergoing CS in SSA.

## Data Availability

The original contributions presented in the study are included in the article/Supplementary Material, further inquiries can be directed to the corresponding author.
